# Radiolabeled 5‑Fluorouracil-Loaded Solid Lipid Nanoparticles: A Potential Platform for Colorectal Cancer Imaging

**DOI:** 10.1021/acsomega.5c06817

**Published:** 2026-01-13

**Authors:** Meliha Eki̇nci̇, İrem Mansuroğlu, Yiğit Uyanikgi̇l, Emel Öykü Çeti̇n Uyanikgi̇l, Derya İlem-Özdemi̇r

**Affiliations:** † Faculty of Pharmacy, Department of Radiopharmacy, 37509 Ege University, 35040 Izmir, Turkiye; ‡ Faculty of Medicine, Department of Histology and Embryology, Ege University, 35040 Izmir, Turkiye; § Faculty of Pharmacy, Department of Pharmaceutical Technology, Department of Biopharmaceutics and Pharmacokinetics, Ege University, 35040 Izmir, Turkiye

## Abstract

Radiolabeled 5-fluorouracil (5-FU)-loaded solid lipid nanoparticles (SLNs) were successfully developed using high shear homogenization and ultrasonication techniques. The SLNs were characterized for particle size, polydispersity index, and zeta potential. The formulations exhibited a mean particle size below 150 nm with narrow size distribution and negative surface charge, indicating good colloidal stability. The radiolabeling of SLNs with technetium-99m ([^99m^Tc]­Tc) was performed using stannous chloride as the reducing agent, achieving a high radiolabeling efficiency (>90%). The *in vitro* radiochemical stability of [^99m^Tc]­Tc-5-FU-SLNs was confirmed in saline, serum, and cell culture medium, where the radiochemical purity remained above 90% for up to 6 h. Partition coefficient studies demonstrated that the radiolabeled formulations exhibited hydrophilic characteristics, supporting their potential for systemic circulation with reduced nonspecific tissue uptake. Cellular binding studies using the HT-29 human colorectal adenocarcinoma cell line revealed that [^99m^Tc]­Tc-5-FU-SLNs demonstrated strong and sustained cell association over time, significantly higher than free [^99m^Tc]­Tc-5-FU and negative controls. The SLN formulations were found to be biocompatible, showing no adverse effects on cell morphology during the incubation period. These findings indicate that radiolabeled 5-FU-loaded SLNs represent a promising platform for potential use in targeted colorectal cancer imaging, pending further *in vivo* validation.

## Graphical abstract

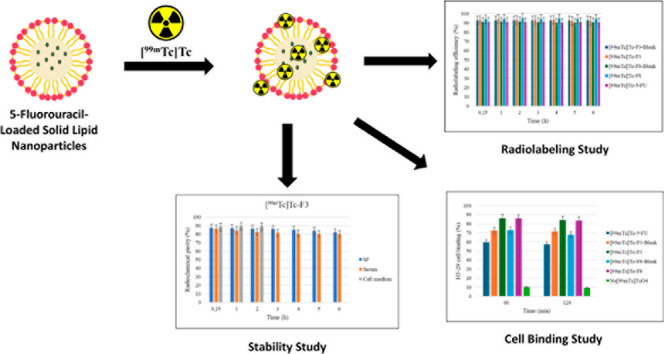

## 1 Introduction

Nuclear medicine plays a critical role in visualizing and quantifying various physiological and functional processes in the human body. This is achieved through the use of radiopharmaceuticals that preferentially accumulate in specific tissues or organs, enabling targeted imaging. The quality of nuclear imaging depends on the selective retention of radiolabeled agents in the target site with minimal background activity. Enhanced image contrast allows for more accurate diagnosis and early detection of pathological conditions.^1,2^

Radiopharmaceuticals are compounds that consist of a radionuclide bound to a bioactive molecule, enabling localization in specific tissues for diagnostic or therapeutic purposes. The bioactive component facilitates selective uptake, while the radionuclide component emits detectable radiation. Among the various radionuclides used in clinical practice, technetium-99m ([^99m^Tc]­Tc) stands out due to its favorable nuclear characteristics, including a short half-life (6 h), optimal gamma emission energy (140 KeV), and ready availability at low cost.^3–5^ Moreover, the diverse oxidation states of [^99m^Tc]Tc (ranging from +7 to −1) provide considerable versatility for complex formation with different ligands, making it an ideal candidate for the development of novel radiopharmaceuticals targeting various organs and pathologies.^6,7^

5-Fluorouracil (5-FU) is a pyrimidine analog widely used in the treatment of a range of malignancies, including colorectal, gastric, breast, and head and neck cancers.^8^ It exerts its antineoplastic effects primarily through the inhibition of thymidylate synthase, thereby disrupting DNA synthesis and inducing cell death. Despite its clinical utility, the therapeutic application of 5-FU is limited by its short plasma half-life, rapid enzymatic degradation, and systemic toxicity, which necessitate frequent administration and often lead to suboptimal drug accumulation at the tumor site.^9^

To address these limitations, novel drug delivery systems such as solid lipid nanoparticles (SLNs) have gained increasing attention.^10–13^ SLNs are biocompatible and biodegradable colloidal carriers that can enhance drug stability, prolong circulation time, and enable controlled release. Their nanoscale size enables passive tumor targeting *via* the enhanced permeability and retention (EPR) effect, which is particularly advantageous for solid tumors such as colorectal cancer. When combined with radiolabeling strategies, SLNs serve as promising platforms for the development of multifunctional agents capable of simultaneous drug delivery and molecular imaging.^14^ ,^15^ However, most previous SLN studies with hydrophilic anticancer agents such as 5-FU have reported moderate encapsulation efficiencies (<80%) and loading capacities (<10%), often requiring surface modifications or additional steps for radiolabeling.^16–24^ In contrast, the present work achieved exceptionally high encapsulation efficiency (>90%) and loading capacity (>18%) for 5-FU without the need for complex chemical modifications, while enabling direct [^99m^Tc]Tc labeling without chelators. This dual capability—high payload incorporation and efficient radiolabeling—combined with strong *in vitro* binding to HT-29 colorectal cancer cells, represents a novel approach not previously described for SLN-based colorectal cancer imaging systems.

Colorectal cancer remains one of the leading causes of cancer-related morbidity and mortality worldwide.^25^ It ranks as the third most commonly diagnosed cancer and the second leading cause of cancer death globally. Early and accurate detection of colorectal malignancies is crucial for improving survival rates, as prognosis significantly worsens with late-stage diagnosis. Conventional imaging modalities often fail to detect small or early stage lesions with sufficient specificity. Therefore, the development of targeted radiopharmaceuticals for colorectal cancer imaging represents a critical area of ongoing research and clinical need.

The aim of this study was to develop and characterize 5-FU loaded SLNs (5-FU-SLNs) and evaluate their potential as targeted imaging agents for colorectal cancer. The formulations were characterized in terms of particle size, zeta potential, polydispersity index (PDI), encapsulation efficiency (EE), loading capacity (LC), and short-term stability. Selected formulations were radiolabeled with [^99m^Tc]­Tc, and radiochemical purity (RP) was evaluated under different reducing agent concentrations using radioactive thin-layer chromatography (RTLC). *In vitro* stability of the radiolabeled 5-FU-SLNs was assessed in saline, serum, and cell culture medium. Finally, cellular binding studies were conducted using HT-29 colorectal adenocarcinoma cells to compare the targeting efficiency of [^99m^Tc]­Tc-5-FU-SLNs with [^99m^Tc]­Tc-SLNs, [^99m^Tc]­Tc-5-FU, and free Na­[^99m^Tc]­TcO_4_.

## 2 Materials and Methods

### 2.1 Materials

Gelucire 48/16 pellets and Dynasan 116 were obtained from Gattefossé SAS (Saint-Priest, France). 5-FU was supplied by Kocak Pharma (Istanbul, Türkiye). Tween 80, Span 80, and Lipoid S100 were purchased from Merck (Darmstadt, Germany). The HT-29 (HTB-38) human colorectal adenocarcinoma cell line was obtained from the American Type Culture Collection (ATCC, Manassas, VA, USA). Sodium pertechnetate (Na­[^99m^Tc]­TcO_4_) was provided by the Department of Nuclear Medicine, Ege University (Izmir, Türkiye).

### 2.2 Spectrophotometric Method for the Quantification of 5-FU

A validated UV–visible spectrophotometric method was established to quantify the amount of 5-FU encapsulated within the SLNs. For this purpose, the UV absorption spectrum of 5-FU was recorded between 200 and 400 nm to determine its maximum absorbance wavelength (λ_max_). Standard solutions of 5-FU at six different concentrations (10, 15, 20, 25, 30, and 40 μg·mL^–1^) were prepared using 0.9% sodium chloride solution (SF) as the solvent.^26^ Based on the absorbance values of these dilutions, a calibration curve was constructed, and the corresponding regression equation and correlation coefficient (*r* ^2^) were calculated to assess the linearity of the method.

### 2.3 Preparation of SLNs and 5-FU-SLNs

SLNs were prepared using a high-speed homogenization followed by ultrasonication method.^27^ In the formulation, Gelucire 48/16 pellets and Dynasan 116 were used as solid lipids; Tween 80, Span 80, and Lipoid S100 served as surfactants; and distilled water constituted the aqueous phase. Initially, the solid lipids were melted by heating them to 5–10 °C above their respective melting points. Simultaneously, the aqueous phase containing surfactants was heated to the same temperature. The hot aqueous phase was then added to the lipid phase, and the mixture was homogenized at 10,000 rpm for 5 min. The resulting emulsion was subjected to ultrasonication at 500 W (20 s cycle for 10 min) and subsequently allowed to cool to room temperature. The prepared SLN formulations were stored in labeled glass vials at 4 °C until further use. The composition of the SLN formulations is summarized in Table 1.

**Table 1:** Composition of Blank SLN Formulations

	Solid Lipids		Surfactants			Aqueous Phase
For. No.	Gelucire 48/16 pellet (mg)	Dynasan 116 (mg)	Tween 80 (mg)	Span 80 (mg)	Lipoid S 100 (mg)	Distilled Water (mL)
F1-Blank	300	-	100	-	-	10
F2-Blank	300	-	-	100	-	10
F3-Blank	300	-	-	-	100	10
F4-Blank	-	300	100	-	-	10
F5-Blank	-	300	-	100	-	10
F6-Blank	-	300	-	-	100	10
F7-Blank	150	150	100	-	-	10
F8-Blank	150	150	-	100	-	10
F9-Blank	150	150	-	-	100	10

Based on the physicochemical characterization results of blank SLNs, formulations exhibiting optimal properties were selected for 5-FU loading. In brief, 1 mg of 5-FU was added to the melted lipid phase (heated to 5–10 °C above its melting point). Subsequently, an aqueous phase containing surfactant was added, and the mixture was homogenized at 10,000 rpm for 5 min. The resulting emulsion was subjected to ultrasonication (500 W, 20 s cycle for 10 min) and cooled to room temperature. The composition of the 5-FU-loaded SLN formulations is presented in Table 2.

**Table 2:** Composition of 5-FU-loaded SLN Formulations

	Solid Lipid		Surfactants		Aqueous Phase	Active Substance
For. No.	Gelucire 48/16 pellet (mg)	Dynasan 116 (mg)	Span 80 (mg)	Lipoid S 100 (mg)	Distilled water (mL)	5-FU (mg)
F3	300	-	-	100	10	1
F8	150	150	100	-	10	1

### 2.4 Characterization of SLNs and 5-FU-SLNs

#### 2.4.1 Mean Particle Size, Polydispersity Index, and Zeta Potential

In this study, the particle size distribution and PDI values of blank and 5-FU-loaded SLN formulations were determined using the dynamic light scattering (DLS) technique with a Malvern Zetasizer Nano ZS 90 (Malvern Instruments, UK).^28^ The measurements were performed at 25 ± 1 °C under ambient conditions. Prior to analysis, SLN samples were diluted appropriately with distilled water to avoid multiple scattering effects. Each measurement was carried out in triplicate, and the average values were reported as mean ± standard deviation.

Zeta potential analysis was conducted to assess the surface charge and colloidal stability of the SLN dispersions. The measurements were performed using a DTS 1060C disposable zeta cuvette equipped with gold electrodes. An electric field of 40 V·cm^–1^ was applied, and electrophoretic mobility was converted into zeta potential values using the Smoluchowski approximation. The measurements were also conducted at room temperature, and three independent replicates were analyzed (*n* = 3).

All obtained data were statistically evaluated and interpreted in relation to formulation composition, with particular emphasis on the influence of lipid type and surfactant combination on particle characteristics.

#### 2.4.2 Encapsulation Efficiency and Drug Loading Capacity

In this study, the encapsulation efficiency (EE %) and loading capacity (LC %) of 5-FU-loaded SLNs were determined using an indirect quantification method based on UV–visible spectrophotometry. Briefly, 1 mL of the 5-FU-SLN dispersion was transferred into a dialysis membrane bag with a molecular weight cutoff of 12–14 kDa. The sealed dialysis bag was subjected to ultracentrifugation at 4750 rpm to separate free (unencapsulated) 5-FU. The supernatant was then filtered through a cellulose nitrate membrane filter to remove residual lipid particles or aggregates. The amount of unencapsulated 5-FU present in the filtrate was quantified using a UV/vis spectrophotometer at the predetermined λ_max_ of 272 nm.

The amount of encapsulated drug was calculated by subtracting the free drug concentration from the initial drug amount added during formulation. EE % and LC % were then calculated using the following equations^29^

$$EE\;\left(\%\right)=\left[\frac{\left(W_{t}-W_{f}\right)}{W_{t}}\right]\times 100$$

$$LC\;\left(\%\right)=\left[\frac{\left(W_{t}-W_{f}\right)}{\left(W_{t}+W_{l}\right)}\right]\times 100$$

*W*_t_ = total amount of 5-FU added (mg). *W*_f_ = amount of free (unencapsulated) 5-FU in supernatant (mg). *W*_l_ = total weight of lipid and excipients (mg).

In this study, the indirect method was preferred due to the hydrophilic nature of 5-FU, which made quantification more feasible in the external aqueous phase. To ensure that no significant drug leakage occurred from the SLNs during the ultracentrifugation and dialysis process, we conducted a control experiment using drug-free SLNs spiked with a known amount of 5-FU. Recovery analysis confirmed that the encapsulated drug remained within the nanoparticles under the applied centrifugation conditions.

All measurements were performed in triplicate, and the results were expressed as mean ± standard deviation.

#### 2.4.3 Scanning Electron Microscope Image

The morphological characteristics of both blank and 5-FU-loaded SLN formulations were evaluated using scanning electron microscopy (SEM). For this purpose, the samples were coated with a thin layer of gold (10 nm) using an EMITECH K550X sputter coater, operated at 15 mA and 6 × 10^–2^ mbar for 1.5 min. Following the coating process, the surface morphology of the formulations was examined using a Philips XL-30S FEG SEM at an accelerating voltage of 7.5 kV and a magnification of 100,000×.

### 2.5 Stability Study of SLNs and 5-FU-SLNs

The stability of the blank and 5-FU-loaded SLN formulations was evaluated under different storage conditions in accordance with International Conference on Harmonisation (ICH) guidelines for stability testing of pharmaceutical products.^30^ The formulations were stored at 4 °C (refrigerated conditions), 25 ± 2 °C/60 ± 5% relative humidity (RH; controlled room temperature), and 40 ± 2 °C/75 ± 5% RH (accelerated conditions) for a period of six months.

At predetermined intervals (0, 3, and 6 months), the samples were withdrawn and analyzed for changes in key physicochemical parameters, including mean particle size, PDI, and zeta potential. The particle size distribution and PDI were measured using DLS (Malvern Zetasizer Nano ZS 90, Malvern Instruments, UK), while zeta potential was determined using electrophoretic light scattering with a DTS 1060C disposable zeta cuvette. All measurements were performed in triplicate and results were reported as mean ± standard deviation.

Changes in these parameters were monitored to assess the physical stability of the blank and 5-FU-loaded SLN formulations over time, as significant variations could indicate aggregation, sedimentation, or loss of colloidal stability.

### 2.6 Radiolabeling of SLNs and 5-FU-SLNs

Both blank and 5-FU-loaded SLN formulations were radiolabeled with [^99m^Tc]Tc using a direct labeling method, in which stannous chloride (SnCl_2_) served as the reducing agent.^31–34^ For the labeling procedure, 0.1 mL of freshly eluted Na­[^99m^Tc]­TcO_4_ solution in isotonic saline (approximately 37 MBq) was added to 1 mL of SLN dispersion. Subsequently, 100 μg of freshly prepared SnCl_2_ solution in distilled water was added to facilitate the reduction of pertechnetate (TcO_4_^–^) to a lower oxidation state that enables binding to the SLN matrix.

To prevent oxidation of Sn^2+^ and ensure efficient radiolabeling, the reaction was conducted under an inert nitrogen atmosphere. The mixture was vortexed for 1 min and incubated at room temperature (25 ± 2 °C) for 5 min, followed by gentle stirring for an additional 10 min to allow complete reduction and incorporation of the radionuclide into the nanoparticles.

Following the labeling, the radiolabeled SLNs were sterilized by filtration through a 0.22 μm polyethersulfone membrane filter (Millipore Corporation, Carrigtwohill, Ireland) to remove potential aggregates and ensure sterility. The filtered formulations were aseptically transferred into sterile evacuated vials and stored at room temperature until further analyses. Radiochemical purity (RP) and in vitro stability of the radiolabeled formulations were evaluated *via* radioactive thin-layer chromatography (RTLC), as detailed in the subsequent sections.

In addition to the radiolabeling of SLN formulations, a control labeling procedure was conducted in which 1 mL of the aqueous 5-FU solution alone was directly labeled with approximately 37 MBq Na­[^99m^Tc]­TcO_4_ in the presence of 100 μg freshly prepared SnCl_2_ under identical conditions.^35^ This control sample served to evaluate the nonspecific binding or direct complexation potential of free 5-FU with [^99m^Tc]Tc in the absence of the lipid carrier system. The labeling process was carried out under a nitrogen atmosphere, followed by incubation and filtration as described for SLNs. The RP (%) of this 5-FU control formulation was also assessed using RTLC to compare its labeling behavior with that of the nanoparticle-bound drug.

#### 2.6.1 Quality Control of Radiolabeled SLNs and 5-FU-SLNs

The RP (%) of the [^99m^Tc]­Tc-labeled blank and 5-FU-loaded SLN formulations was evaluated to determine both the efficiency of the radiolabeling process and the in vitro stability of the radiolabeled products over time. RP is a key indicator reflecting the proportion of radioactivity that remains stably bound to the nanoparticulate system, as opposed to undesirable species such as free pertechnetate (Na­[^99m^Tc]­TcO_4_) or hydrolyzed-reduced technetium (^99m^TcO_2_).^5^

RTLC was employed for RP analysis, using ITLC-SG (silica gel-coated glass fiber) strips as the stationary phase. Two different mobile phases were utilized to discriminate among radiolabeled SLNs, free pertechnetate, and hydrolyzed technetium species:

- Acetone: where Na­[^99m^Tc]­TcO_4_ migrates with the solvent front (*R*_f_ ≈ 1.0), while radiolabeled SLNs and ^99m^TcO_2_ remain at the origin (*R*_f_ ≈ 0.0).
- Pyridine/acetic acid/water (3:5:1.5, v/v/v): where both Na­[^99m^Tc]­TcO_4_ and the labeled SLNs migrate, while ^99m^TcO_2_ remains at the origin.

After chromatographic development, the distribution of radioactivity on the strips was quantified using a radio-TLC scanner (Bioscan AR-2000, USA). RP (%) was calculated using the following equation^36^

RP(%)=100−[%freeTc‐99m+%hydrolizedTc‐99m]

In this formula, while % free ^99m^Tc corresponds to radioactivity attributed to unbound Na­[^99m^Tc]­TcO_4_, % hydrolyzed ^99m^Tc corresponds to radioactivity attributed to colloidal ^99m^TcO_2._

RP was assessed at predefined time intervals up to 6 h postlabeling to evaluate the stability of the radiolabeled nanoparticles *in vitro*. Each measurement was performed in triplicate, and results were expressed as mean ± standard deviation.

#### 2.6.2 *In Vitro* Stability of Radiolabeled SLNs and 5-FU-SLNs

The *in vitro* stability of the [^99m^Tc]­Tc-labeled blank and 5-FU-loaded SLN formulations was investigated to evaluate the integrity of the radiolabel over time under various physiological and ambient conditions. Stability assessment is essential to ensure that the radionuclide remains firmly associated with the nanoparticle matrix without significant dissociation, which could lead to nonspecific background activity and reduced imaging accuracy *in vivo*.^37^

For this purpose, 100 μL of each freshly prepared radiolabeled SLN formulation was incubated under three different conditions:

1 In isotonic saline at 25 °C for up to 6 h to simulate ambient storage stability.
2 In a 1:1 (v/v) mixture of phosphate-buffered saline (PBS) and fetal bovine serum (FBS) at 37 °C for 6 h to replicate extended physiological exposure.
3 In complete cell culture medium (Dulbecco’s modified Eagle medium [DMEM] supplemented with 10% FBS) at 37 °C for 2 h to mimic short-term exposure to intracellular conditions.

At predetermined time points (0, 1, 2, 3, 4, 5, and 6 h, depending on the condition), aliquots were withdrawn and analyzed using ITLC-SG, as previously described in Section 2.6.1. RP % was calculated by quantifying the fraction of radioactivity associated with intact radiolabeled nanoparticles relative to the total radioactivity, including free and hydrolyzed [^99m^Tc]­Tc.

Stability was defined as maintaining RP ≥ 90% throughout the incubation period.^38^ This study design enabled the assessment of radiolabel retention under both simulated biological and ambient conditions, providing insight into the expected *in vivo* performance and storage stability of the radiolabeled SLN formulations.

#### 2.6.3 Partition Coefficient Study of Radiolabeled SLNs and 5-FU-SLNs

The partition coefficient (log *P*) of the [^^99^m^Tc]­Tc-labeled blank and 5-FU-loaded SLN formulations was determined to assess the lipophilicity of the radiolabeled nanoparticles, a critical factor influencing their biodistribution, cellular uptake, and membrane permeability.

The log *P* value was calculated based on the distribution of radioactivity between a lipophilic organic phase (*n*-octanol) and a hydrophilic aqueous phase (phosphate-buffered saline, PBS; pH 7.4). Briefly, 500 μL of n-octanol and 500 μL of PBS were placed in a 1.5 mL Eppendorf tube, followed by the addition of 50 μL of the radiolabeled SLN formulation. The mixture was vortexed for 1 min to ensure homogeneous mixing and then centrifuged at 5000 rpm for 30 min at room temperature to facilitate phase separation. Following centrifugation, the upper (*n*-octanol) and lower (PBS) phases were carefully separated without cross-contamination. A 100 μL aliquot from each phase was collected, and the radioactivity was measured using a gamma counter. The partition coefficient was calculated using the equation:

$$\log P=\log\left(\frac{radio\;activity\;in\;n‐octanol\;phase}{radio\;activity\;in\;PBS\;phase}\right)$$

Each experiment was performed in triplicate to ensure reproducibility, and results were expressed as mean ± standard deviation. A positive log *P* indicates a higher affinity of the radiolabeled formulation for the lipophilic phase, while a negative log *P* suggests a preference for the hydrophilic phase.

### 2.7 Cell Culture Study

HT-29 human colorectal adenocarcinoma cells were cultured in DMEM supplemented with 10% FBS and 0.5 mg·mL^–1^ l-glutamine/penicillin–streptomycin solution. The cells were maintained in 25 cm^2^ tissue culture flasks under standard incubation conditions (37 °C, 5% CO_2_, and humidified atmosphere). Subculturing was performed at 80–90% confluency, and cells were seeded into 6-well plates at a density of 2 × 10^6^ cells per well for further experiments.

#### 2.7.1 Transepithelial Electrical Resistance Measurement

The integrity and tightness of the cell monolayer were evaluated using transepithelial electrical resistance (TEER) measurements with an epithelial volt-ohm meter (EVOM, World Precision Instruments). Measurements were taken before and after the experiment (*n* = 6) to assess cell viability and barrier function. The TEER value (Ω·cm^2^) was calculated using the following equation^31^

$$TEER\;value=\left(R_{monolayer}-R_{blank}\right)\times A$$

*R*_blank_ = resistance of the cell-covered membrane. *R*_monolayer_ = resistance of the blank membrane (without cells). *A* = surface area of the membrane (cm^2^).

#### 2.7.2 Cell Binding Study

The cellular uptake of [^99m^Tc]­Tc-labeled SLN formulations was evaluated in HT-29 cells to determine their binding affinity. Radiolabeled SLN formulations (∼37 MBq) were diluted in complete culture medium and added to the cells. Incubations were carried out at 37 °C for predetermined time intervals (60 and 120 min) to assess time-dependent cellular interaction.

After incubation, the supernatants were aspirated and collected. Cells were washed sequentially with 1 mL of fresh culture medium and 1 mL of phosphate-buffered saline (PBS, pH 7.4) to remove unbound particles. To detach the cells, 0.5 mL of trypsin–EDTA was added, and the resulting cell suspensions were transferred into separate tubes.

The radioactivity of the cell-associated fraction (bound) and that of control samples (formulations incubated without cells) was measured using a gamma counter (Hidex, Finland). The cell binding percentage (CB %) was calculated using the following equation

$$CB\;\left(\%\right)=\left[\frac{radio\;activity\;in\;cells}{radio\;activity\;in\;control}\right]$$

### 2.8 Statistical Analysis

All experimental data were obtained from at least three independent replicates (*n* ≥ 3), and the results were expressed as mean ± standard deviation (SD). Statistical analyses were performed to assess differences between experimental groups and to evaluate the significance of the findings. A *p*-value of less than 0.05 was considered indicative of statistical significance. All statistical analyses were performed using SPSS software (version 31, IBM Corp., Armonk, NY, USA) or equivalent statistical analysis software. Graphical representations and data plots were generated using GraphPad Prism (version 10.5.0, GraphPad Software, San Diego, CA, USA) to aid data visualization.

## 3 Results & Discussion

### 3.1 Spectrophotometric Method for the Quantification of 5-FU

A UV–visible spectrophotometric method was developed and validated for the quantitative determination of 5-FU encapsulated within the SLNs. This method was employed for the indirect calculation of EE % and LC % by measuring the concentration of free (unencapsulated) 5-FU present in the supernatant after separation of the nanoparticles.

The spectrophotometric analysis was performed using a UV/vis spectrophotometer (Shimadzu UV-1800, Shimadzu Corporation, Kyoto, Japan) across a wavelength range of 200–400 nm. The maximum absorption wavelength (λ_max_) of 5-FU in saline was determined to be 272 nm (Figure 1A). All subsequent quantitative measurements were conducted at this wavelength to ensure optimal sensitivity and accuracy.

**Figure 1:**
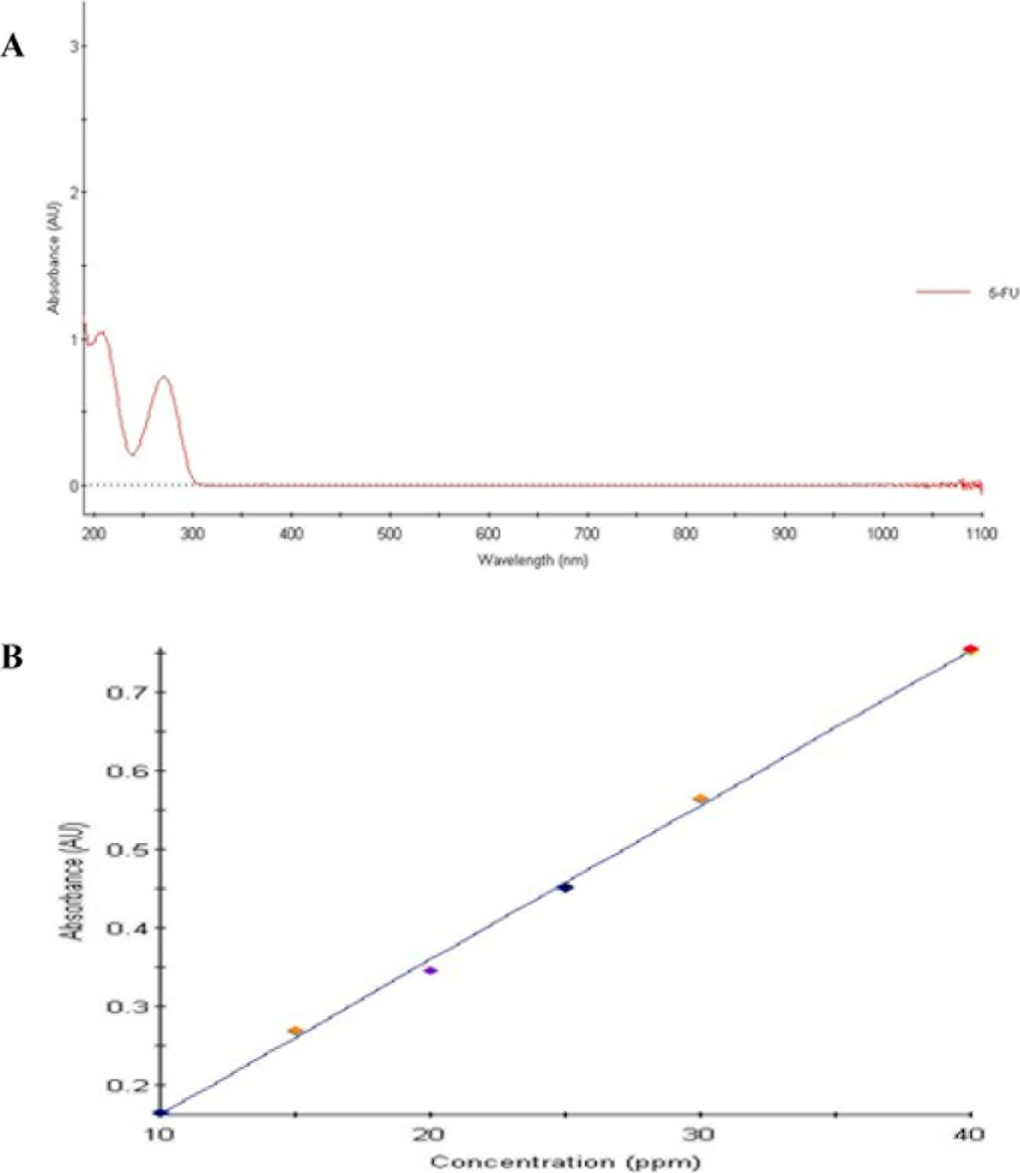
(A) UV spectrum of 5-FU showing the λ_max_ at 272 nm in saline, (B) calibration curve of 5-FU in saline (10–40 μg·mL^–1^), linear regression equation: *y* = 0.02*x* – 0.04, *r* ^2^ = 0.99835.

A calibration curve was established by preparing standard solutions of 5-FU at six different concentrations (10, 15, 20, 25, 30, and 40 μg·mL^–1^) in saline. The absorbance of each standard solution was measured at 272 nm, and the calibration curve was generated by plotting absorbance values against concentration. The resulting linear regression equation was *y* = 0.02*x* – 0.04, with a correlation coefficient (*r* ^2^) of 0.99835, indicating excellent linearity within the tested concentration range (Figure 1B).

### 3.2 Preparation, Characterization and Stability of SLNs and 5-FU-SLNs

The physicochemical characterization of both blank and 5-FU-loaded SLNs is critical for evaluating their potential in drug delivery and radiopharmaceutical applications. Parameters such as mean particle size, PDI, and zeta potential serve as key indicators of colloidal stability, size homogeneity, and surface charge, all of which significantly affect *in vivo* performance, including systemic circulation, biodistribution, tumor accumulation *via* the EPR effect, and cellular uptake efficiency.^39,40^

In the present study, SLNs were successfully prepared using a combination of high-speed homogenization and ultrasonication. The resulting formulations exhibited a wide range of physicochemical profiles, with particle size being notably influenced by the type and ratio of lipid and surfactant components (Table 3). Among the blank formulations, F8-Blank demonstrated the smallest particle size (100.1 ± 1.36 nm) with moderate uniformity (PDI 0.423 ± 0.043), whereas F9-Blank exhibited an exceptionally large size (1155 ± 94.75 nm) and high polydispersity (PDI 0.970 ± 0.040), suggesting its instability and unsuitability for further development. F3-Blank (114.0 ± 1.70 nm, PDI 0.418 ± 0.040) also showed favorable particle size and monodispersity, making it a promising candidate for drug encapsulation and passive targeting applications.

**Table 3:** Physicochemical Properties of SLNs

Formulations	Particle Size (nm ± ss)	PDI (± ss)	Zeta Potential (mV ± ss)
F1-Blank	327.0 ± 16.69	0.350 ± 0.012	–9.22 ± 4.25
F2-Blank	189.1 ± 2.899	0.429 ± 0.001	–36.8 ± 7.09
F3-Blank	**114.0** ± **1.701**	**0.418** ± **0.040**	**–10.3** ± **1.18**
F4-Blank	265.5 ± 1.710	0.370 ± 0.051	–18.9 ± 0.96
F5-Blank	214.3 ± 13.72	0.296 ± 0.047	–40.0 ± 3.95
F6-Blank	127.2 ± 5.091	0.475 ± 0.041	–24.6 ± 2.46
F7-Blank	156.8 ± 3.452	0.472 ± 0.045	–15.2 ± 1.05
F8-Blank	**100.1** ± **1.363**	**0.423** ± **0.043**	**–15.4** ± **0.00**
F9-Blank	1155 ± 94.75	0.970 ± 0.040	–21.0 ± 1.64

Zeta potential values of the formulations ranged from −9.22 mV to −40.0 mV. Notably, F2-Blank, F5-Blank, and F6-Blank displayed zeta potentials exceeding the −30 mV threshold, typically associated with electrostatically stable colloidal systems. However, even formulations with moderately negative zeta potentials (e.g., F3 and F8) retained sufficient surface charge to ensure short- to medium-term physical stability under standard storage and biological conditions. The negative surface charges observed across all formulations are attributable to the inherent characteristics of the selected lipid and surfactant materials, which promote electrostatic repulsion and prevent aggregation.^41^

Upon encapsulation of 5-FU into the selected SLN formulations, both F3 and F8 retained their nanoscale size characteristics, demonstrating particle diameters of 133.6 ± 6.86 nm and 130.9 ± 0.21 nm, respectively (Table 4). The narrow size distributions, especially evident in F3 with a remarkably low PDI of 0.108 ± 0.001, indicate a high degree of homogeneity and suggest reliable manufacturing reproducibility. The maintenance of negative surface charge after drug loading (−22.8 ± 0.87 mV for F3 and −16.5 ± 0.74 mV for F8) supports the continued electrostatic stabilization of the nanoparticulate systems, which is critical for preventing aggregation, promoting colloidal stability, and enhancing *in vivo* dispersion performance.

**Table 4:** Physicochemical Properties of 5-FU-SLNs

Formulations	Particle Size (nm ± ss)	PDI (± ss)	Zeta Potential (mV ± ss)	EE (%)	LC (%)
F3	133.6 ± 6.859	0.108 ± 0.001	–22.8 ± 0.866	90.12 ± 2.4	18.4 ± 1.8
F8	130.9 ± 0.212	0.195 ± 0.037	–16.5 ± 0.735	91.06 ± 0.7	20.1 ± 1.5

The notable reduction in PDI observed in F3 and F8 after 5-FU loading may be attributed to the interaction of the drug with the lipid matrix, which likely facilitated a more ordered internal structure during nanoparticle formation. The presence of 5-FU may have increased the viscosity of the lipid phase or influenced surfactant dynamics, promoting the formation of more uniform particles during homogenization and ultrasonication.

In terms of drug incorporation, both SLN formulations exhibited remarkably high EE % and LC %, exceeding 90% and 18%, respectively. These elevated values indicate successful entrapment of the hydrophilic 5-FU molecule within the lipid matrix and minimal drug loss during the production process. The superior entrapment may be attributed to the optimized formulation conditions and the use of a dual lipid phase in F8 (combining Gelucire 48/16 and Dynasan 116), which likely provided a more structured and organized lipid core for efficient drug loading.

Such efficient drug incorporation is particularly advantageous for radiopharmaceutical applications, where high payload retention and consistent delivery are critical.^42^ The ability of both F3 and F8 to encapsulate over 90% of the drug and achieve LC values above 18% places them among the most effective SLN systems reported to date for hydrophilic drugs. Comparable EE % values have been previously described in lipid-based carriers, but the LC % achieved here surpasses those in most similar studies, further validating the design strategy employed in this work.^43^

Morphological analysis using SEM (Figure 2) confirmed the spherical shape and smooth surface architecture of both blank and 5-FU-loaded nanoparticles. The particles appeared discrete, nonaggregated, and consistent in size, aligning well with DLS results. Notably, the F8 formulation (Figure 2D) exhibited a uniform particle population with diameters consistent with the measured nanoscale range (∼100–130 nm), providing visual confirmation of the formulation’s monodispersity. These morphological features are characteristic of well-formed SLNs and corroborate previous reports describing lipid-based nanocarriers with similar geometric profiles.^44^

**Figure 2:**
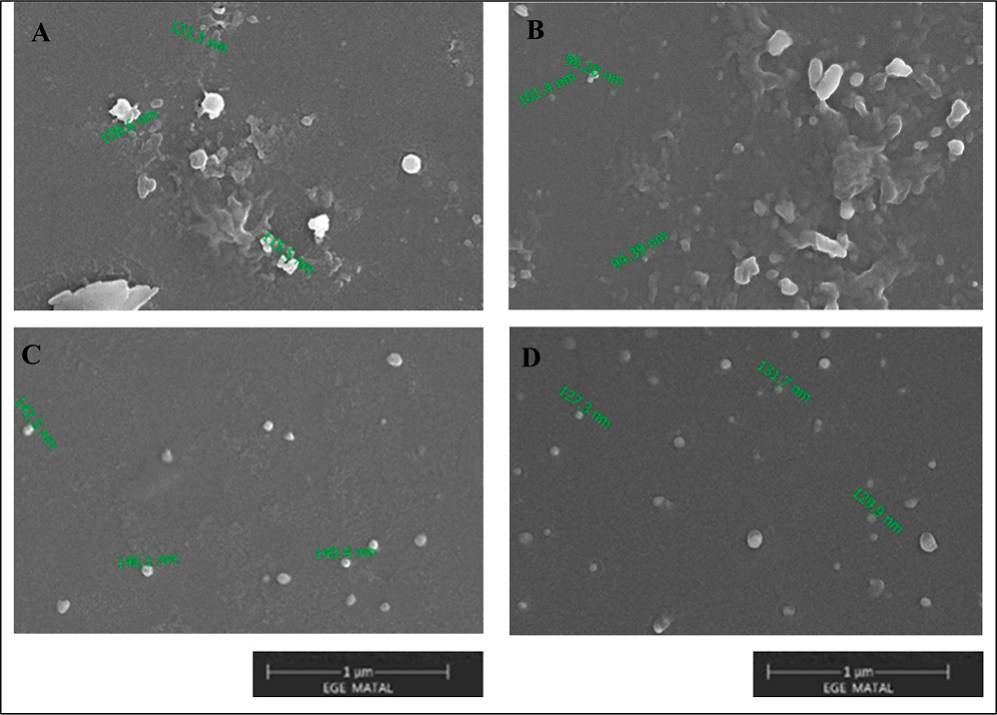
SEM images of (A) F3-Blank, (B) F8-Blank, (C) 5-FU-loaded F3, and (D) 5-FU-loaded F8 formulations, illustrating the spherical morphology and nonaggregated structure of the solid lipid nanoparticles. All images were acquired at 100,000× magnification under 10.00 kV using a Philips XL-30S FEG SEM after gold coating.

Stability assessments conducted under ICH-recommended conditions demonstrated that both F3 and F8 formulations—whether blank or 5-FU-loaded—retained their physicochemical properties over six months of storage (Table 5). Only minimal and gradual increases in particle size (typically ≤ 10%) and modest variations in PDI were observed, particularly under accelerated conditions (40 °C/75% RH). Such changes remained within acceptable colloidal stability limits, with no evidence of phase separation, sedimentation, or particle aggregation. Moreover, zeta potential values remained consistently negative throughout the storage period, reinforcing the persistence of electrostatic stabilization mechanisms.

**Table 5:** Stability Results of SLNs under Different Storage Conditions

		4 °C			25 ± 2 °C/60 ± 5% relative humidity			40 ± 2 °C/75 ± 5% relative humidity		
Formulations	Time (month)	Particle Size (nm ± ss)	PDI (± ss)	Zeta Potential (mV ± ss)	Particle Size (nm ± ss)	PDI (± ss)	Zeta Potential (mV ± ss)	Particle Size (nm ± ss)	PDI (± ss)	Zeta Potential (mV ± ss)
F3-Blank	**0**	114.0 ± 1.70	0.418 ± 0.040	–10.3 ± 1.18	114.0 ± 1.70	0.418 ± 0.040	–10.3 ± 1.18	114.0 ± 1.70	0.418 ± 0.040	–10.3 ± 1.18
	**3**	116.2 ± 2.10	0.422 ± 0.045	–10.1 ± 1.20	117.5 ± 2.50	0.425 ± 0.050	–10.0 ± 1.25	118.0 ± 2.80	0.430 ± 0.055	–9.8 ± 1.30
	**6**	117.0 ± 2.30	0.425 ± 0.050	–10.0 ± 1.22	120.1 ± 3.00	0.430 ± 0.055	–9.7 ± 1.28	123.0 ± 3.50	0.435 ± 0.060	–9.5 ± 1.35
F3	**0**	133.6 ± 6.86	0.108 ± 0.001	–22.8 ± 0.87	133.6 ± 6.86	0.108 ± 0.001	–22.8 ± 0.87	133.6 ± 6.86	0.108 ± 0.001	–22.8 ± 0.87
	**3**	135.1 ± 7.10	0.110 ± 0.002	–22.5 ± 0.90	136.8 ± 7.50	0.113 ± 0.003	–22.2 ± 0.95	138.5 ± 7.90	0.115 ± 0.004	–22.0 ± 0.98
	**6**	136.0 ± 7.20	0.112 ± 0.003	–22.3 ± 0.92	139.5 ± 7.80	0.115 ± 0.004	–22.0 ± 0.97	142.0 ± 8.10	0.118 ± 0.005	–21.7 ± 1.00
F8-Blank	**0**	100.1 ± 1.36	0.423 ± 0.043	–15.4 ± 0.00	100.1 ± 1.36	0.423 ± 0.043	–15.4 ± 0.00	100.1 ± 1.36	0.423 ± 0.043	–15.4 ± 0.00
	**3**	101.5 ± 1.50	0.425 ± 0.045	–15.2 ± 0.20	103.0 ± 1.80	0.428 ± 0.050	–15.0 ± 0.25	105.0 ± 2.00	0.430 ± 0.055	–14.8 ± 0.30
	**6**	102.5 ± 1.60	0.428 ± 0.050	–15.0 ± 0.22	105.5 ± 2.10	0.430 ± 0.055	–14.7 ± 0.28	108.5 ± 2.50	0.435 ± 0.060	–14.5 ± 0.35
F8	**0**	130.9 ± 0.21	0.195 ± 0.037	–16.5 ± 0.74	130.9 ± 0.21	0.195 ± 0.037	–16.5 ± 0.735	130.9 ± 0.21	0.195 ± 0.037	–16.5 ± 0.74
	**3**	132.0 ± 0.35	0.197 ± 0.040	–16.3 ± 0.75	134.5 ± 0.50	0.200 ± 0.045	–16.0 ± 0.78	137.0 ± 0.70	0.205 ± 0.050	–15.7 ± 0.80
	**6**	133.0 ± 0.40	0.200 ± 0.043	–16.2 ± 0.76	136.5 ± 0.60	0.205 ± 0.050	–15.8 ± 0.79	140.0 ± 0.90	0.210 ± 0.055	–15.5 ± 0.82

The sustained physical stability can be attributed to the strategic selection and synergy of lipid and surfactant components. Previous studies have highlighted the role of cosurfactants such as Lipoid S100 and Span 80 in enhancing nanoparticle stability by improving interfacial coverage and reducing surface tension over time.^45^ This is consistent with the current findings, wherein both F3 and F8 formulations, incorporating these surfactants, maintained their integrity under various storage conditions.

Collectively, the high drug loading efficiency, favorable particle size, uniformity, surface morphology, and long-term stability of F3 and F8 strongly support their potential as robust nanocarrier systems for 5-FU delivery. Their physicochemical robustness and stability also make them promising candidates for further radiolabeling and *in vitro/in vivo* evaluation as colorectal cancer imaging agents.

A number of previous studies have investigated 5-FU-loaded nanoparticulate systems, particularly focusing on SLNs and liposomal carriers, utilizing a variety of lipid matrices, surfactants, and formulation methods to improve drug entrapment efficiency, colloidal stability, and particle size distribution. For instance, Kazi et al. (2019) developed folate-peptide conjugated 5-FU nanoparticles with a particle size of 118 ± 3 nm, an EE % of 67.4 ± 1.8%, and a LC % of 3.7 ± 0.4%, demonstrating spherical morphology under SEM analysis.^16^ Similarly, Bahadur et al. (2020) prepared SLNs using stearic acid and Poloxamer 188, reporting a particle size of 210.6 ± 2.4 nm, zeta potential of −28.6 ± 1.2 mV, EE % of 72.4 ± 3.1%, and LC % of 4.9 ± 0.3%, indicating moderate colloidal stability.^17^

In another formulation study, Dodia and Shirsat (2021) utilized glyceryl monostearate and Tween 80 to produce 5-FU-loaded SLNs with a particle size of 180.4 ± 5.6 nm, PDI of 0.272, zeta potential of −21.5 ± 0.9 mV, EE % of 68.9 ± 2.7%, and LC % of 5.6 ± 0.4%.^18^ Likewise, Garg et al. (2014) developed stearic acid–based SLNs with 172.4 ± 6.2 nm size, 72.3 ± 2.1% EE, and 4.8 ± 0.5% LC.^19^ Cosurfactant systems have also been explored; for example, in a recent study by Ali et al. (2023), poloxamer 188 and lecithin-based SLNs exhibited a particle size of 189.3 ± 3.2 nm and an EE % of 75.4 ± 1.8%.^20^ Yassin et al. (2010) reported even larger particles (∼278 nm), with EE % of 72.8% and LC % of 9.5%,^21^ while Smith et al. (2020) achieved EE % of 84.6 ± 1.4% and LC % of 8.72 ± 0.3% using Compritol 888 ATO and Poloxamer 188.^22^

Additional studies, such as Amasya et al. (2016), reported SLNs with 157.3 ± 4.2 nm size, 72.9 ± 3.1% EE, and 6.45 ± 0.5% LC.^23^ Yalçın et al. (2021) applied active loading methods in liposomal systems, resulting in an EE % of 84.9 ± 2.6%, significantly higher than conventional liposomal techniques.^24^

When compared with these previously reported systems, the F3 and F8 SLN formulations developed in the present study demonstrate superior performance. Both formulations maintained nanoscale sizes (133.6 ± 6.86 nm for F3 and 130.9 ± 0.21 nm for F8) and favorable homogeneity (PDI 0.108 and 0.195, respectively). More notably, the EE % values exceeded 90% (90.12 ± 2.4% for F3 and 91.06 ± 0.7% for F8), and LC % values were remarkably high (18.4 ± 1.8% for F3 and 20.1 ± 1.5% for F8), significantly surpassing those reported in the literature, where LC % typically ranged between 3% and 9%.

Furthermore, both formulations exhibited moderately negative zeta potentials (−22.8 ± 0.87 mV for F3 and −16.5 ± 0.74 mV for F8), ensuring sufficient colloidal stability. These findings, reinforced by long-term stability under ICH storage conditions, position F3 and F8 SLNs as promising carriers for 5-FU delivery, especially in radiopharmaceutical applications targeting colorectal cancer.

### 3.3 Radiolabeling, Quality Control, *In Vitro* Stability and Partition Coefficient Study of SLNs and 5-FU-SLNs

Radiolabeling studies confirmed that both blank and 5-FU-loaded SLN formulations were successfully labeled with [^99m^Tc]Tc using a direct labeling approach, in which SnCl_2_ served as the reducing agent. All formulations achieved high radiolabeling efficiencies (≥90%) within 15 min at room temperature, as shown in Figure 3. This finding underscores the practicality of direct radiolabeling for lipid-based nanosystems, consistent with prior studies utilizing [^99m^Tc]­Tc-labeled liposomes and polymeric nanoparticles.^31–33^

**Figure 3:**
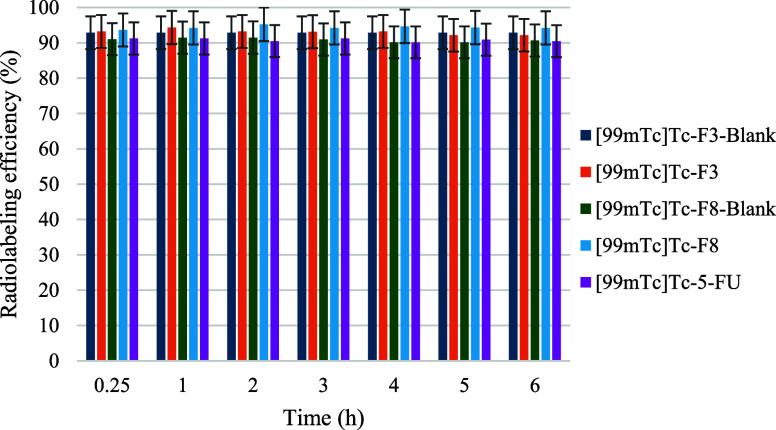
Radiolabeling efficiency (%) of [^99m^Tc]­Tc-5-FU-SLNs and [^99m^Tc]­Tc-5-FU.

This method circumvents the need for bifunctional chelators such as DTPA or MAG_3_, which are commonly employed in radiopharmaceutical chemistry but introduce additional synthetic steps. Smith et al. (2020) similarly reported >90% labeling efficiency for [^99m^Tc]­Tc-labeled SLNs formulated with Compritol 888 ATO,^22^ while Amasya et al. (2016) demonstrated efficient labeling of stearic acid–based SLNs for hydrophilic drug encapsulation.^23^

In the present study, a control sample composed of free 5-FU solution labeled under identical conditions—without any lipid carrier—exhibited a comparatively lower radiochemical purity (∼90%) and reduced stability over time. This result highlights the critical role of the SLN matrix in stabilizing the reduced technetium species, minimizing hydrolysis or dissociation, and facilitating sustained radiolabel retention.

Our previous work, in which a 5-FU derivative was directly radiolabeled with [^99m^Tc]­Tc, showed adequate but comparatively lower radiochemical performance than the SLN-based formulations reported.^35^ Taken together, these findings support the interpretation that [^99m^Tc]Tc primarily associates with the lipid/surfactant matrix—providing coordination sites and a protective microenvironment—rather than binding strongly to 5-FU itself, which explains the higher radiochemical purity and stability achieved with SLNs.

RP % is a critical quality parameter that reflects the percentage of total radioactivity associated with the desired radiolabeled nanoparticle, free from unbound pertechnetate (Na­[^99m^Tc]­TcO_4_) and hydrolyzed technetium species (^99m^TcO_2_). In this study, RP % was assessed using ITLC-SG with two complementary mobile phases: acetone, which separates free pertechnetate (*R*_f_ ≈ 1.0), and pyridine/acetic acid/water (3:5:1.5, v/v/v), which separates hydrolyzed technetium species (*R*_f_ ≈ 0.0) from radiolabeled nanoparticles.

As presented in Figure 4, the RTLC chromatograms confirmed clear separation of species and high RP % for all [^99m^Tc]­Tc-labeled SLN formulations. Throughout the 6 h observation period, RP % values consistently exceeded 90%, aligning with the minimum quality criteria established by the IAEA and FDA for radiopharmaceuticals intended for diagnostic use.^38^

**Figure 4:**
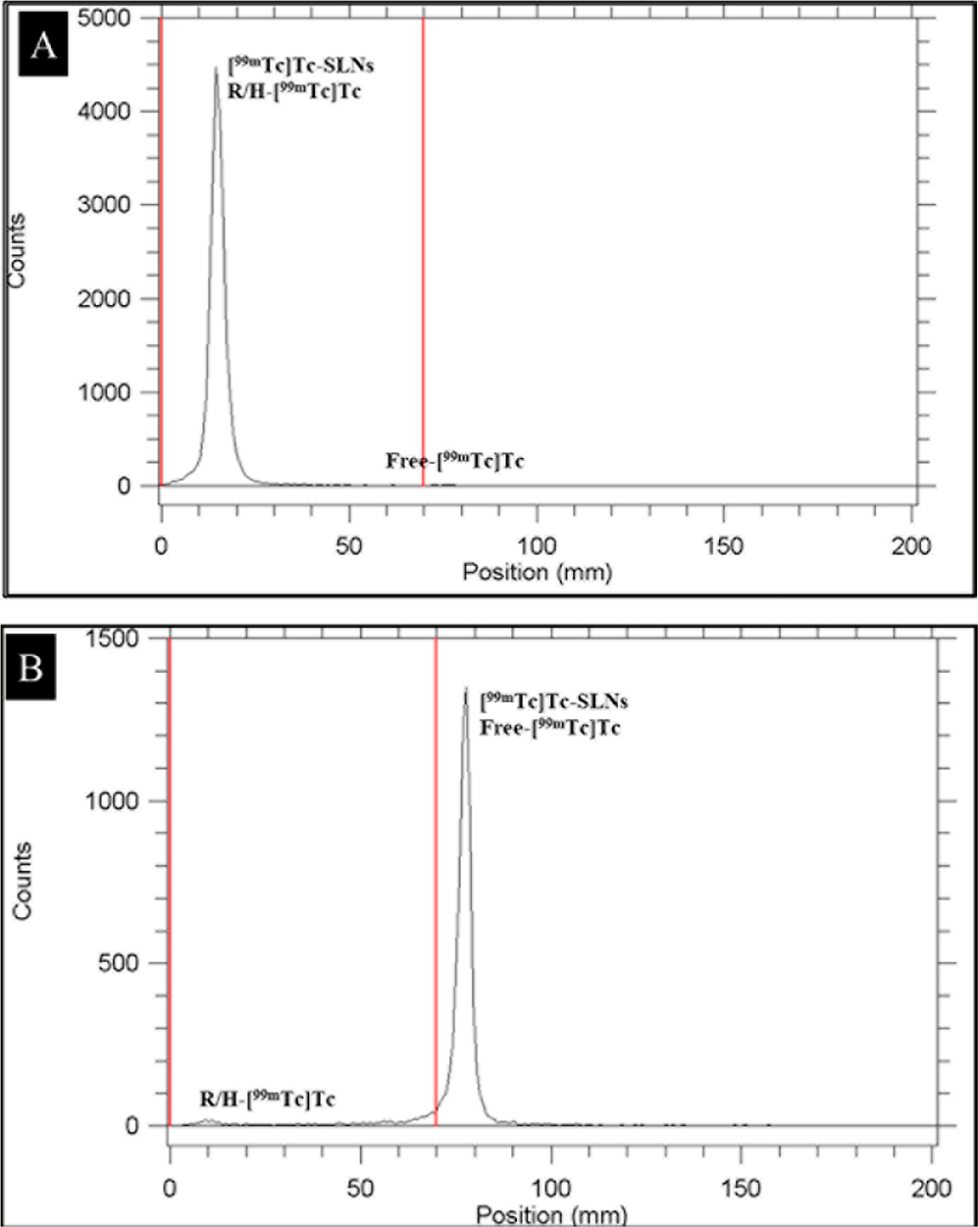
RTLC chromatograms of [^99m^Tc]­Tc-5-FU-SLN formulations in (A) acetone and (B) pyridine/acetic acid/water, demonstrating separation of free ^99m^Tc, hydrolyzed ^99m^TcO_2_, and radiolabeled SLNs.

Similar RP % levels were previously reported by Patel et al. (2014), who radiolabeled 5-FU-loaded SLNs with [^99m^Tc]Tc and attributed the high purity to effective interactions between the lipid matrix and reduced technetium species.^19^ This interaction appears to stabilize the radiolabeled complex while minimizing the formation of ^99m^TcO_2_ and Na­[^99m^Tc]­TcO_4_.

The present findings further validate the robustness of stannous chloride-mediated direct labeling strategies for nanocarrier systems. The absence of significant colloidal or unbound impurities supports the structural stability of the radiolabeled complexes. These outcomes are also in accordance with previous studies involving [^99m^Tc]­Tc-labeled lipid-based or polymeric nanoparticles, which demonstrated reliable RP stability suitable for in vivo imaging applications.^16,23^

The *in vitro* stability of the radiolabeled SLN formulations was evaluated under various physiological and ambient conditions, including incubation in saline at 25 °C, a 1:1 (v/v) mixture of PBS and FBS at 37 °C, and complete cell culture medium (DMEM supplemented with 10% FBS) at 37 °C (Figure 5). Across all conditions, both blank and 5-FU-loaded SLNs exhibited high RP %, consistently ≥90% throughout the designated incubation periods (6 h for saline and serum, 2 h for DMEM). These findings indicate strong association of [^99m^Tc]Tc with the SLN matrix and minimal radiolabel dissociation or degradation, fulfilling IAEA and FDA criteria for radiopharmaceutical stability.^38^

**Figure 5:**
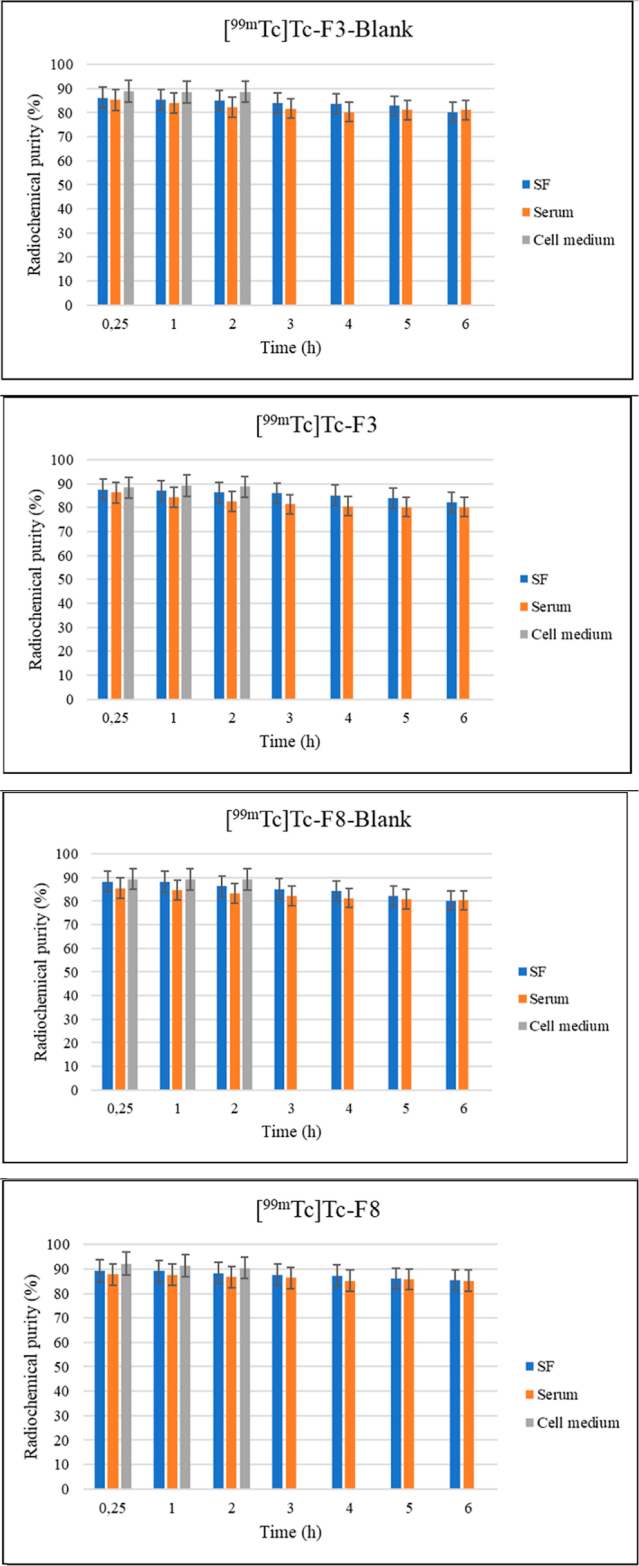
*In vitro* radiochemical stability profiles of [^99m^Tc]­Tc-labeled SLN formulations in different media. The RP % of [^99m^Tc]­Tc–F3–Blank, [^99m^Tc]­Tc–F3, [^99m^Tc]­Tc–F8–Blank, and [^99m^Tc]­Tc–F8 were evaluated in saline, serum, and cell culture medium over a 6 h period. All formulations maintained RP % ≥ 90%, indicating high radiolabel stability under simulated physiological conditions.

Minor reductions in RP % were noted under serum and DMEM conditions, particularly in [^99m^Tc]­Tc-5-FU and [^99m^Tc]­Tc–F8 formulations. These declines are likely due to enzymatic degradation and protein binding interactions in complex biological fluids, which can compromise the stability of unprotected radiolabels. Nevertheless, all formulations maintained RP within acceptable limits for diagnostic imaging.

These observations are consistent with earlier reports. For instance, Smith et al. (2020) reported >90% stability for [^99m^Tc]­Tc-labeled SLNs in serum-containing media,^22^ and Yassin et al. (2014) demonstrated that SLNs effectively protect technetium labels from hydrolysis in biological fluids.^21^ In contrast, directly labeled [^99m^Tc]­Tc-5-FU (Figure 6) without a carrier exhibited significantly reduced RP %, particularly in serum, emphasizing the critical role of the lipid nanoparticle in stabilizing the radiolabel and preventing premature release.

**Figure 6:**
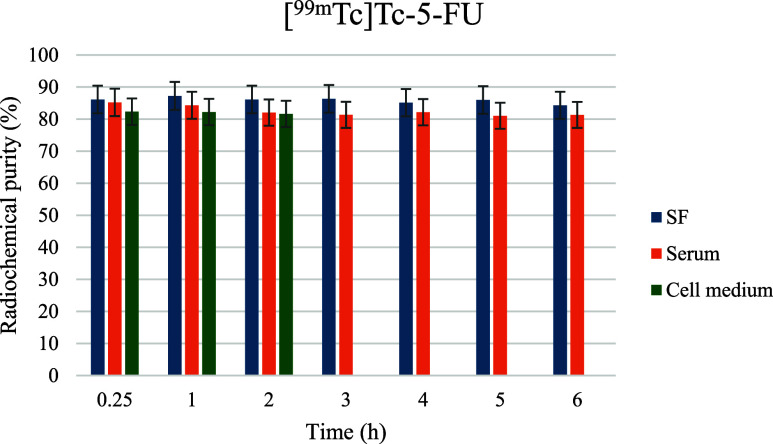
Radiochemical stability profile of directly labeled [^99m^Tc]­Tc-5-FU in different incubation media. RP % was monitored over 6 h in saline, serum, and cell culture medium.

Lipophilicity is a critical parameter influencing the biodistribution, cellular uptake, and pharmacokinetics of radiopharmaceuticals, offering valuable insight into the expected in vivo behavior of nanoparticulate systems.^46^ The partition coefficients (log P) of the [^99m^Tc]­Tc-labeled formulations ranged from −1.884 ± 0.028 for [^99m^Tc]­Tc–F3–Blank to −0.408 ± 0.063 for [^99m^Tc]­Tc-5-FU, as shown in Table 6. All formulations exhibited negative log P values, indicative of hydrophilic characteristics, which are desirable for diagnostic agents aiming to avoid nonspecific tissue accumulation and promote renal clearance.

**Table 6:** Log P Values of [^^99^m^Tc]­Tc-Labeled SLN Formulations

Formulations	Log *P* values
[^99m^Tc]Tc-5-FU	–0.408 ± 0.063
[^99m^Tc]Tc-F3-Blank	–1.884 ± 0.028
[^99m^Tc]Tc-F3	–1.514 ± 0.032
[^99m^Tc]Tc-F8-Blank	–1.018 ± 0.055
[^99m^Tc]Tc-F8	–1.154 ± 0.167
Na[^99m^Tc]TcO_4_	–0.823 ± 0.152

The most hydrophilic formulations, [^99m^Tc]­Tc–F3–Blank and [^99m^Tc]­Tc–F3, demonstrated the lowest log *P* values, suggesting enhanced aqueous solubility and improved circulation potential, while reducing hepatic uptake. In contrast, [^99m^Tc]­Tc-5-FU, lacking a lipid carrier system, showed the least negative log *P*, consistent with the intrinsic polarity of 5-FU and a higher likelihood of rapid renal elimination. The intermediate log *P* values observed for [^99m^Tc]­Tc–F8 and [^99m^Tc]­Tc–F8–Blank formulations indicate a more balanced hydrophilic–lipophilic profile, which may optimize both blood circulation and cellular uptake.

These findings are consistent with previous reports in the literature. For instance, Pandey et al. (2020) demonstrated that SLN-based delivery systems significantly enhance aqueous dispersion and minimize nonspecific tissue uptake due to their hydrophilic surface properties.^17^ Similarly, Kazi et al. (2019) reported that folate-peptide conjugated 5-FU nanoparticles with moderate hydrophilicity achieved improved passive accumulation in tumor tissues, highlighting the relevance of controlled lipophilicity for targeted delivery.^16^ In line with these observations, Amasya et al. (2016) showed that hydrophilic nanocarriers radiolabeled with [^99m^Tc]Tc displayed prolonged systemic circulation, reduced hepatic retention, and improved imaging contrast.^23^ Collectively, these studies support the premise that [^99m^Tc]­Tc-labeled SLNs with negative log *P* values are well-suited for diagnostic applications due to their favorable biodistribution and pharmacokinetic profiles.

Several recent studies have explored lipid-based nanocarriers for cancer imaging applications, such as liposomal nanohybrids incorporating gold nanorods for NIR-triggered theranostics,^47^ liposomal platforms integrating quantum dots or metallic nanoparticles for multimodal bioimaging,^48^ biodegradable fluorescent liposomal nanohybrids for photodriven tumor diagnosis and therapy,^49^ and multifunctional liposomal systems combining gold nanoparticles and graphene quantum dots for combined imaging and therapy.^50^ While these systems demonstrate impressive multifunctionality, they often involve complex preparation steps, multiple functionalization stages, and sometimes low drug loading efficiencies. In contrast, the SLNs developed in this study achieve exceptionally high 5-FU encapsulation efficiency (>90%) and loading capacity (>18%) without additional chemical modifications and enable direct [^99m^Tc]Tc labeling without chelators. This streamlined design offers both simplicity in formulation and strong *in vitro* binding to colorectal cancer cells, positioning our system as a promising and efficient alternative for potential theranostic applications.

### 3.4 Cell Culture Study of Radiolabeled SLNs and 5-FU-SLNs

To evaluate the impact of radiolabeled SLN formulations on cellular monolayer integrity, TEER values were measured before and after the experiments using HT-29 colorectal cancer cells (Table 7). Initial TEER values ranged from 1242 ± 35 to 1268 ± 28 Ω·cm^–2^, while postincubation values were recorded between 1258 ± 38 and 1284 ± 30 Ω·cm^–2^, indicating negligible variation during the 2 h exposure period.

**Table 7:** TEER Values of HT-29 Cell Line During the Experimental Period (*n* = 6) (*p* < 0.05)

	TEER value (Ω·cm^–2^)	
Time (min)	Before Experiment (Ω·cm^–2^)	After Experiment (Ω·cm^–2^)
0	1242 ± 35	1258 ± 38
60	1256 ± 30	1272 ± 36
120	1268 ± 28	1284 ± 30

Importantly, no significant drop in TEER values was observed (*p* < 0.05), and all fluctuations remained within the accepted physiological threshold of <40% variance.^51^ These data confirm that neither the radiolabeled formulations nor the incubation process adversely impacted the structural integrity or viability of the HT-29 cell monolayers.

These results are consistent with Mansoori et al. (2020), who reported that surface-modified nanocarriers preserve epithelial barrier function and exhibit favorable safety profiles *in vitro*.^52^ Similarly, Kamel et al. (2017) showed that chitosan-coated SLNs did not compromise HT-29 cell monolayer resistance, suggesting that the nanoparticle matrix is biocompatible and suitable for epithelial delivery.^53^

The cellular binding efficiencies of the [^99m^Tc]­Tc-labeled SLN formulations were evaluated on HT-29 human colorectal adenocarcinoma cells at two time points (60 and 120 min) to investigate their potential for selective tumor targeting. As illustrated in Figure 7, at 60 min, [^99m^Tc]­Tc–F3 and [^99m^Tc]­Tc–F8 demonstrated the highest cellular binding percentages (∼85–90%), indicating efficient and rapid interaction with the cancer cell surface. Their corresponding blank formulations ([^99m^Tc]­Tc–F3–Blank and [^99m^Tc]­Tc–F8–Blank) also exhibited strong binding (75–85%), suggesting that the SLN matrix -comprising lipid type and surfactant composition- plays a critical role in facilitating cell–nanoparticle interactions, even in the absence of drug loading. This strong binding can be attributed to the optimized nanoparticle size, surface hydrophilicity, and colloidal stability -parameters known to enhance cell–nanoparticle interactions.

**Figure 7:**
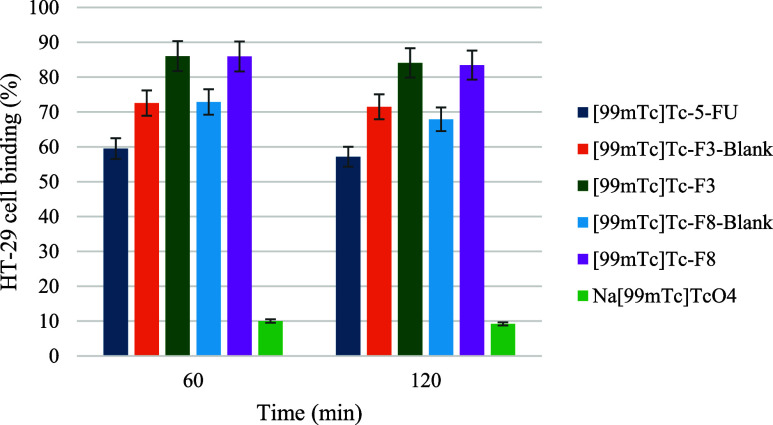
HT-29 cell binding efficiencies (%) of [^99m^Tc]­Tc-labeled formulations and control (Na­[^99m^Tc]­TcO_4_) at 60 and 120 min (*p* < 0.05).

At 120 min, binding percentages remained stable or increased slightly across all SLN formulations, confirming their sustained interaction with the cell surface. This trend may be attributed to the colloidal stability, hydrophilic surface properties, and nanoscale particle size of the SLNs, all of which contribute to effective adhesion or internalization by HT-29 cells.

In contrast, [^99m^Tc]­Tc-5-FU exhibited lower binding efficiency (∼60% at 60 min), with minimal enhancement over time. This reduced interaction is primarily attributed to the small molecular size and hydrophilic nature of 5-FU, which limits its passive membrane association and cellular uptake. Similar findings were reported by Kamel et al. (2017), who observed significantly lower cellular retention of free 5-FU compared to its SLN-encapsulated form in colon cancer cells.^53^ These results highlight the critical role of SLNs as delivery vehicles, not only for enhancing physicochemical stability but also for improving the interaction of therapeutic agents with target cells. Furthermore, the negative control, Na­[^99m^Tc]­TcO_4_, demonstrated minimal binding (∼10%), thereby confirming that the cellular association of radiolabeled SLNs and [^99m^Tc]­Tc-5-FU is specific and not a consequence of nonspecific adsorption or electrostatic interactions.

Although the present study demonstrated strong and sustained association of [^99m^Tc]­Tc-labeled SLNs with HT-29 colorectal cancer cells, the cellular uptake mechanism was not specifically investigated. As the formulations in this study were not functionalized with targeting ligands, the observed interaction is most likely mediated by passive processes, such as nonspecific adsorption and endocytosis facilitated by the nanoparticles’ physicochemical properties (size, surface charge, and hydrophilicity). Future studies will focus on elucidating the precise uptake pathways—distinguishing between passive and receptor-mediated mechanisms—through the use of specific inhibitors and competitive binding assays.

The present findings highlight that [^99m^Tc]­Tc-labeled SLNs can serve not only as imaging agents but also as a multifunctional nanosystem with potential therapeutic applications. Recent advances have demonstrated that nanosystems can be engineered to promote tumor therapy via multiple interactions, including combinational drug delivery, tumor microenvironment modulation, and synergistic therapeutic strategies.^54–56^ Incorporating such multi-interaction strategies in future designs could enhance the dual diagnostic and therapeutic potential of the developed SLN platform.

## 4 Conclusion

This study successfully demonstrated the development, radiolabeling, physicochemical characterization, and in vitro evaluation of [^99m^Tc]­Tc-labeled SLNs, both blank and 5-FU-loaded, as potential diagnostic agents for colorectal cancer imaging. Direct radiolabeling using stannous chloride resulted in high labeling efficiency (≥90%) within 15 min under ambient conditions, eliminating the need for bifunctional chelators and simplifying the radiolabeling process. Quality control analyses via RTLC confirmed high RP and minimal presence of free or hydrolyzed technetium species across all formulations.

In vitro stability studies conducted under physiologically relevant conditions (saline, serum, and culture medium) revealed that the radiolabeled SLNs maintained radiochemical integrity for up to 6 h, with RP % values consistently above 90%, indicating strong radiolabel retention and minimal dissociation. Partition coefficient (log *P*) analysis demonstrated that all formulations exhibited hydrophilic character, a desirable feature for prolonged blood circulation and reduced nonspecific tissue accumulation in diagnostic imaging applications.

Cell binding studies performed on HT-29 human colorectal cancer cells further confirmed the potential of the SLNs for targeted imaging. Among the tested formulations, [^99m^Tc]­Tc–F3 and [^99m^Tc]­Tc–F8 showed the highest cellular binding (∼85–90%), while their corresponding blank SLNs also exhibited strong binding efficiencies (∼75–85%), suggesting that the nanoparticle matrix alone significantly contributes to cell–nanoparticle interaction. In contrast, free [^99m^Tc]­Tc-5-FU demonstrated limited binding (∼60%), emphasizing the importance of nanoparticle encapsulation in enhancing cellular association and retention. The negligible binding observed with Na­[^99m^Tc]­TcO_4_ confirmed the specificity of SLN-mediated interactions.

Despite these encouraging outcomes, several limitations should be acknowledged. The study was confined to *in vitro* conditions, and the *in vivo* behavior of the radiolabeled SLNs—including their pharmacokinetics, biodistribution, imaging performance, and safety profile—remains to be evaluated in suitable animal models. Additionally, cellular interaction studies were restricted to a single cancer cell line; broader screening across different tumor and healthy cell types would provide a more comprehensive understanding of the formulation’s targeting specificity. While *in vitro* media mimicked physiological environments, they do not fully replicate the complexity of biological systems, including enzymatic degradation, immune response, and clearance mechanisms that could influence nanoparticle performance *in vivo*. Furthermore, *in vivo* imaging studies could not be performed due to financial constraints and the unavailability of a gamma camera system at the current research facility, limiting the full translational evaluation of the developed formulations.

In conclusion, the [^99m^Tc]­Tc-labeled SLNs developed in this study demonstrated excellent radiolabeling efficiency, high *in vitro* stability, and strong cellular binding to HT-29 colorectal cancer cells. The optimized F3 and F8 formulations, in particular, showed great promise for selective tumor targeting. These results highlight the potential of SLNs as effective and biocompatible carriers for radiodiagnostic applications. Overall, the study lays a solid foundation for further *in vivo* investigations toward clinical translation in colorectal cancer imaging.

## References

[1] Ilem-Ozdemir, D. , Gundogdu, E. A. , Ekinci, M. , Ozgenc, E. , Asikoglu, M. Nuclear medicine and radiopharmaceuticals for molecular diagnosis. Biomedical Applications of Nanoparticles; Içinde, Grumezescu, A. M. , Eds., 1st ed.; Elsevier Inc., 2019; p 457.

[2] Kowalsky, R. J. , Falen, S. W. Radiopharmaceuticals in Nuclear Pharmacy and Nuclear Medicine, 3rd ed.; American Pharmacists Association (APhA), 2013; p 875.

[3] Payolla F. B., Massabni A. C., Orvig C. (2019). Radiopharmaceuticals for diagnosis
in nuclear medicine: A short review. Eclét.
Quím..

[4] Duatti A. (2021). Review on
99mTc radiopharmaceuticals with emphasis on new advancements. Nucl. Med. Biol..

[5] Boschi A., Uccelli L., Martini P. (2019). A picture
of modern Tc-99m radiopharmaceuticals:
Production, chemistry, and applications in molecular imaging. Appl. Sci..

[6] Costa B., Ilem-Özdemir D., Santos-Oliveira R. (2019). Technetium-99m metastable radiochemistry
for pharmaceutical applications: old chemistry for new products. J. Coord. Chem..

[7] Lever S. Z., Lever J. R. (2009). Technetium-99m pharmaceuticals: Preparation
and quality
control in nuclear medicine. J. Nucl. Med..

[8] Blondy S., David V., Verdier M., Mathonnet M., Perraud A., Christou N. (2020). 5-Fluorouracil resistance
mechanisms
in colorectal cancer: From classical pathways to promising processes. Cancer Sci..

[9] Valencia-Lazcano A. A., Hassan D., Pourmadadi M., Shamsabadipour A., Behzadmehr R., Rahdar A., Medina D. I., Díez-Pascual A. M. (2023). 5-Fluorouracil
nano-delivery systems as a cutting-edge for cancer therapy. Eur. J. Med. Chem..

[10] Luo W. C., Lu X. (2023). Solid Lipid Nanoparticles
for Drug Delivery. Methods Mol. Biol..

[11] Montoto S. S., Muraca G., Ruiz M. E. (2020). Solid Lipid
Nanoparticles for Drug
Delivery: Pharmacological and Biopharmaceutical Aspects. Front. Mol. Biosci..

[12] Mukherjee S., Ray S., Thakur R. S. (2009). Solid lipid
nanoparticles: A modern formulation approach
in drug delivery system. Indian J. Pharm. Sci..

[13] Sastri K. T., Radha G. V., Pidikiti S., Vajjhala P. (2020). Solid lipid nanoparticles:
Preparation techniques, their characterization, and an update on recent
studies. J. Appl. Pharm. Sci..

[14] Ekinci M., Ilem-Ozdemir D., Gundogdu E., Asikoglu M. (2015). Methotrexate loaded
chitosan nanoparticles: Preparation, radiolabeling and in vitro evaluation
for breast cancer diagnosis. J. Drug Deliv.
Sci. Technol..

[15] Ekinci M., Koksal-Karayildirim C., Ilem-Ozdemir D. (2023). Radiolabeled methotrexate loaded
chitosan nanoparticles as imaging probe for breast cancer: Biodistribution
in tumor-bearing mice. J. Drug Deliv. Sci. Technol..

[16] Kazi J., Mukhopadhyay R., Sen R., Jha T., Ganguly S., Debnath M. C. (2019). Design of 5-fluorouracil (5-FU) loaded,
folate conjugated
peptide linked nanoparticles, a potential new drug carrier for selective
targeting of tumor cells. Medchemcomm.

[17] Pandey A. N., Rajpoot K., Jain S. K. (2021). 5-Fluorouracil
Loaded Orally Administered
WGA-decorated Poly­(lacticco- glycolic Acid) Nanoparticles for Treatment
of Colorectal Cancer: In Vivo Evaluation. Curr.
Nanomed..

[18] Dodia R., Shirsat M. (2021). 5 Fluorouracil Solid
Lipid Nanoparticles (SLNs), Formulation
and Evaluation for the treatment of Skin Disorders. Nat. Volatiles Essent. Oils.

[19] Patel M. N., Lakkadwala S., Majrad M. S., Injeti E. R., Gollmer S. M., Shah Z. A., Boddu S. H. S., Nesamony J. (2014). Characterization and
Evaluation of 5-Fluorouracil-Loaded Solid Lipid Nanoparticles Prepared
via a Temperature-Modulated Solidification Technique. Ageing Int..

[20] Ali A., Madni A., Shah H., Jamshaid T., Jan N., Khan S., Khan M. M., Mahmood M. A. (2023). Solid lipid-based
nanoparticulate system for sustained release and enhanced in-vitro
cytotoxic effect of 5-fluorouracil on skin Melanoma and squamous cell
carcinoma. PLoS One.

[21] Yassin A. E. B., Anwer M. K., Mowafy H. A., El-Bagory I. M., Bayomi M. A., Alsarra I. A. (2010). Optimization of
5-fluorouracil solid-lipid
nanoparticles: A preliminary study to treat colon cancer. Int. J. Med. Sci..

[22] Smith T., Affram K., Nottingham E. L., Han B., Amissah F., Krishnan S., Trevino J., Agyare E. (2020). Application of smart
solid lipid nanoparticles to enhance the efficacy of 5-fluorouracil
in the treatment of colorectal cancer. Sci.
Rep..

[23] Amasya G., Badilli U., Aksu B., Tarimci N. (2016). Quality by design case
study 1: Design of 5-fluorouracil loaded lipid nanoparticles by the
W/O/W double emulsion - Solvent evaporation method. Eur. J. Pharm. Sci..

[24] Yalçin T. E., Yetgin C., Yilmaz A. (2021). Development
of 5-fluorouracil-loaded
nano-sized liposomal formulation by two methods: Strategies to enhance
encapsulation efficiency of hydrophilic drugs. J. Res. Pharm..

[25] Siegel R. L., Giaquinto A. N., Jemal A. (2024). Cancer statistics, 2024. CA Cancer J. Clin..

[26] Ekinci M., Akbaba H., Santos-Oliveira R., Ilem-Ozdemir D. (2022). Development
and validation of UV/VIS spectroscopy method for determination of
atezolizumab in pharmaceutical products. Exp.
Biomed. Res..

[27] Musielak E., Feliczak-Guzik A., Nowak I. (2022). Optimization of the Conditions of
Solid Lipid Nanoparticles (SLN) Synthesis. Molecules.

[28] Ekinci M., Yeǧen G., Aksu B., İlem-Özdemir D. (2022). Preparation
and Evaluation of Poly­(lactic acid)/Poly­(vinyl alcohol) Nanoparticles
Using the Quality by Design Approach. ACS Omega.

[29] Kanwar R., Gradzielski M., Mehta S. K. (2018). Biomimetic Solid Lipid Nanoparticles
of Sophorolipids Designed for Antileprosy Drugs. J. Phys. Chem. B.

[30] Branch S. K. (2005). Guidelines
from the International Conference on Harmonisation (ICH). J. Pharm. Biomed. Anal..

[31] Ekinci M., Çalışkan E. E., Çakar B., İlem-Özdemir D., Uyanıkgil Y., Uyanıkgil E. O. ¨. C. (2023). [99mTc]­Technetium-labeled niosomes:
Radiolabeling, quality control, and in vitro evaluation. ACS Omega.

[32] Ekinci M., Öztürk A. A., Santos-Oliveira R., İlem-Özdemir D. (2022). The use of Lamivudine-loaded PLGA
nanoparticles in the diagnosis of lung cancer: Preparation, characterization,
radiolabeling with 99mTc and cell binding. J.
Drug Deliv. Sci. Technol..

[33] Ekinci M., Dos Santos C. C., Alencar L. M. R., Akbaba H., Santos-Oliveira R., Ilem-Ozdemir D. (2022). Atezolizumab-Conjugated Poly­(lactic acid)/Poly­(vinyl
alcohol) Nanoparticles as Pharmaceutical Part Candidates for Radiopharmaceuticals. ACS Omega.

[34] Banerjee I., De K., Chattopadhyay S., Bandyopadhyay A. K., Misra M. (2014). An easy and effective method for
radiolabelling of solid lipid nanoparticles. J. Radioanal. Nucl. Chem..

[35] Ilem-Ozdemir D., Atlihan-Gundogdu E., Ekinci M., Halay E., Ay K., Karayildirim T., Asikoglu M. (2019). Radiolabeling and in vitro evaluation
of a new 5-fluorouracil derivative with cell culture studies. J. Label. Compd. Radiopharm..

[36] Ekinci M., Alencar L. M. R., Lopes A. M., Santos-Oliveira R., İlem-Özdemir D. (2023). Radiolabeled Human Serum Albumin
Nanoparticles Co-Loaded with Methotrexate and Decorated with Trastuzumab
for Breast Cancer Diagnosis. J. Funct. Biomater..

[37] Mei L., Chu T. (2011). Technetium-99m radiopharmaceutical chemistry. Prog. Chem..

[38] Zolle, I. Technetium-99m Pharmaceuticals: Preparation and Quality Control in Nuclear Medicine; Springer, 2007; pp 1–345.

[39] Qushawy M., Nasr A. (2020). Solid lipid nanoparticles (SLNs) as nano drug delivery carriers:
Preparation, characterization and application. Int. J. Appl. Pharm..

[40] Kanojia N., Sharma N., Gupta N., Singh S. (2022). Applications
of nanostructured
lipid carriers: Recent advancements and patent review. Biointerface Res. Appl. Chem..

[41] Modena M. M., Rühle B., Burg T. P., Wuttke S. (2019). Nanoparticle
Characterization:
What to Measure?. Adv. Mater..

[42] Garud A., Singh D., Garud N. (2012). Solid Lipid Nanoparticles
(SLN):
Method, Characterization and Applications. Int.
Curr. Pharm. J..

[43] Danaei M., Dehghankhold M., Ataei S., Hasanzadeh
Davarani F., Javanmard R., Dokhani A., Khorasani S., Mozafari M. R. (2018). Impact of Particle Size and Polydispersity Index on
the Clinical Applications of Lipidic Nanocarrier Systems. Pharmaceutics.

[44] Honary S., Zahir F. (2013). Effect of Zeta Potential
on the Properties of Nano-Drug Delivery
Systems - A Review (Part 2). Trop. J. Pharm.
Res..

[45] Jaiswal S., Gupta G. D. (2013). Recent advances
in solid lipid nanoparticles and challenges. Indo Am. J. Pharm. Res..

[46] Dhande R., Tyagi A., Sharma R. K., Thakkar H. (2017). Biodistribution
study
of 99mTc-gemcitabine-loaded spherulites in Sprague–Dawley rats
by gamma scintigraphy to investigate its lung targeting potential. J. Microencapsul..

[47] Chauhan D. S., Prasad R., Devrukhkar J., Selvaraj K., Srivastava R. (2018). Disintegrable
NIR Light Triggered Gold Nanorods Supported Liposomal Nanohybrids
for Cancer Theranostics. Bioconjug. Chem..

[48] Kannan R., Prabakaran P., Basu R., Pindi C., Senapati S., Muthuvijayan V., Prasad E. (2019). Mechanistic Study on the Antibacterial
Activity of Self-Assembled Poly­(aryl ether)-Based Amphiphilic Dendrimers. ACS Appl. Bio Mater..

[49] Prasad R., Chauhan D. S., Yadav A. S., Devrukhkar J., Singh B., Gorain M., Temgire M., Bellare J., Kundu G. C., Srivastava R. (2018). A biodegradable fluorescent nanohybrid
for photo-driven tumor diagnosis and tumor growth inhibition. Nanoscale.

[50] Prasad R., Jain N. K., Yadav A. S., Chauhan D. S., Devrukhkar J., Kumawat M. K., Shinde S., Gorain M., Thakor A. S., Kundu G. C. (2020). Liposomal nanotheranostics for multimode targeted
in vivo bioimaging and near-infrared light mediated cancer therapy. Commun. Biol..

[51] Elitez Y., Ekinci M., Ilem-Ozdemir D., Gundogdu E., Asikoglu M. (2018). Tc-99m radiolabeled
alendronate sodium microemulsion: Characterization and permeability
studies across caco-2 cells. Curr. Drug Deliv..

[52] Mansoori B., Mohammadi A., Abedi-Gaballu F., Abbaspour S., Ghasabi M., Yekta R. (2020). Hyaluronic
acid-decorated liposomal
nanoparticles for targeted delivery of 5-fluorouracil into HT-29 colorectal
cancer cells. J. Cell. Physiol..

[53] Kamel K. M., Khalil I. A., Rateb M. E., Elgendy H., Elhawary S. (2017). Chitosan-Coated
Cinnamon/Oregano-Loaded Solid Lipid Nanoparticles to Augment 5-Fluorouracil
Cytotoxicity for Colorectal Cancer: Extract Standardization, Nanoparticle
Optimization, and Cytotoxicity Evaluation. J.
Agric. Food Chem..

[54] Chiang M., Hsu C., Pan W., Tran N., Lee Y., Chiang W., Liu Y. C., Chen Y. W., Chiou S. H., Hu S. H. (2025). Reprogramming
Dysfunctional Dendritic Cells by a Versatile Catalytic Dual Oxide
Antigen-Captured Nanosponge for Remotely Enhancing Lung Metastasis
Immunotherapy. ACS Nano.

[55] Huynh T. M. H., Huang P.-X., Wang K.-L., Tran N.-T., Iao H. M., Pan W.-C., Chang Y. H., Lien H. W., Lee A. Y. L., Chou T. C. (2025). Reprogramming Immunodeficiency
in Lung
Metastases via PD-L1 siRNA Delivery and Antigen Capture of Nanosponge-Mediated
Dendritic Cell Modulation. ACS Nano.

[56] Moorthy T., Chen C.-K., Liu Z.-H., Yalamandala B. N., Huynh T. M. H., Iao H. M., Pan W. C., Lien H. W., Lee A. Y. L., Chiang W. H. (2025). Wireless
chargeable
gold Yarnball-mediated mitochondrial depolarization for dendritic
cell detainment in programmed brain tumor immunotherapy. Nano Today. Aralık.

